# Predicting the unexpected total fertilization failure in conventional *in vitro* fertilization cycles: What is the role of semen quality?

**DOI:** 10.3389/fcell.2023.1133512

**Published:** 2023-02-23

**Authors:** Xiaoming Jiang, Jiali Cai, Lanlan Liu, Zhenfang Liu, Jinhua Chen, Chao Yang, Kaijie Chen, Xiaolian Yang, Jie Geng, Caihui Ma, Shuiyan Lian, Li Xu, Jianzhi Ren

**Affiliations:** ^1^ Reproductive Medicine Center, Xiamen University Affiliated Chenggong Hospital, Xiamen, Fujian, China; ^2^ School of Medicine, Xiamen University, Xiamen, Fujian, China

**Keywords:** total fertilization failure, semen parameters, oocyte yield, poor responder, prediction model

## Abstract

**Background:** Male and female gametes factors might contribute to the total fertilization failure (TFF). In first *in vitro* fertilization (IVF) cycles, decision-making of insemination protocol was mainly based on semen quality for the contribution of female clinical characteristics to TFF remained obscure. The purpose of the study was to evaluate the role of semen quality in predicting unexpected TFF.

**Methods:** A single-center retrospective cohort analysis was performed on 19539 cycles between 2013 and 2021. Two algorithms, a Least Absolute Shrinkage and Selection Operator (LASSO) and an Extreme Gradient Boosting (Xgboost) were used to create models with cycle characteristics parameters. By including semen parameters or not, the contribution of semen parameters to the performance of the models was evaluated. The area under the curve (AUC), the calibration, and the net reclassification index (NRI) were used to evaluate the performance of the models.

**Results:** The prevalence of TFF were .07 (95%CI:0.07-0.08), and .08 (95%CI:0.07-0.09) respectively in the development and validation group. Including all characteristics, with the models of LASSO and Xgboost, TFF was predicted with the AUCs of .74 (95%CI:0.72-0.77) and .75 (95%CI:0.72-0.77) in the validation group. The AUCs with models of LASSO and Xgboost without semen parameters were .72 (95%CI:0.69-0.74) and .73 (95%CI:0.7-0.75). The models of LASSO and Xgboost with semen parameters only gave the AUCs of .58 (95%CI:0.55-0.61) and .57 (95%CI:0.55-0.6). For the overall validation cohort, the event NRI values were −5.20 for the LASSO model and −.71 for the Xgboost while the non-event NRI values were 10.40 for LASSO model and 0.64 for Xgboost. In the subgroup of poor responders, the prevalence was .21 (95%CI:0.18-0.24). With refitted models of LASSO and Xgboost, the AUCs were .72 (95%CI:0.67-0.77) and .69 (95%CI:0.65-0.74) respectively.

**Conclusion:** In unselected patients, semen parameters contribute to limited value in predicting TFF. However, oocyte yield is an important predictor for TFF and the prevalence of TFF in poor responders was high. Because reasonable predicting power for TFF could be achieved in poor responders, it may warrant further study to prevent TFF in these patients.

## Introduction

While there is a trend of increasing use of intracytoplasmic sperm injection (ICSI), the conventional *in vitro* fertilization (IVF) practice remained an important part of contemporary assisted reproductive technology (ART) practices (Adamson et al., 2018), resulting in an average fertilization rate of 76% (ESHRE Special Interest Group of Embryology and Alpha Scientists in Reproductive Medicine, 2017). However, unexpected total fertilization failure (TFF) still occurred in 5%–20% of IVF treatment cycles (Combelles et al., 2010; Huang et al., 2015). The TFF refers to the failure of all retrieved mature oocytes to form two pronuclear zygotes after insemination in 15–18 h (Shinar et al., 2014). With the TFF, no embryos were available for transfer and the treatments would be canceled. That would incur distressful emotions and excess financial burden for the repeated medical treatment cycles for infertile couples.

ICSI is proven to increase fertilization rates when TFF has previously occurred with conventional insemination (Practice, 2020). Therefore, identifying the potential clinical risk factors and establishing predicting models might help choose the appropriate insemination protocol for patients with high risks of TFF. A few studies have attempted to predict the risks of fertilization failure among patients receiving conventional IVF (Repping et al., 2002; Krog et al., 2015; Li J. et al., 2021; Tian et al., 2022), demonstrating discriminatory powers as the form of AUCs ranging from .72 to .80. However, they still lack a consensus on the key predictors for TFF. For instance, Repping et al. (2002) achieved a notable AUC of .72 with total motile sperm count (TMC) alone while Tian et al. (2022) included a comprehensive set of predictors to report a similar AUC of .74 and the sperm quality diagnoses rather than TMC was used in their model. In addition, the lack of external validation and heterogeneity of the outcomes measured may limit the applicability of these models in clinical practice.

In the first cycle of ART, the decision for selecting ICSI over conventional IVF in patients without absolute indications (Babayev et al., 2014) is generally based on the semen parameters measured during the infertility workup or on the day of ovum pickup (OPU) (Li J. et al., 2021; Bjorndahl et al., 2022; Dcunha et al., 2022). However, the criteria are often arbitrary. While patients with less than 2 million motile spermatozoa might be recommended for ICSI (Dcunha et al., 2022), some authors propose more rigorous criteria (Repping et al., 2002). The work of Repping et al. (2002) might suggest that increasing the minimal TMC requirement for conventional IVF could effectively prevent TFF. Paradoxically, however, TFF may also occur in the conventional IVF treatment cycles with normal semen parameters (Wang and Swerdloff, 2014; Esteves et al., 2017). It is known that female gamete quality might also contribute to TFF (ESHRE Special Interest Group of Embryology and Alpha Scientists in Reproductive Medicine, 2017). However, unlike the male factor, the contribution of female clinical characteristics to TFF remains obscure. As current evidence does not support the use of ICSI solely according to the female characteristics, such as female age and poor response (Practice, 2020), evaluating the relative importance of male and female characteristics in the prediction of TFF may facilitate the decision making on the selection of insemination protocol in the first ART cycle.

In the present study, we retrospectively analyzed 19539 cycles that received their first IVF treatments in our center from January 2013 to December 2021. The aim was to evaluate the value of basic semen parameters in predicting the occurrence of TFF and try to create models to predict unexpected TFF for the patients receiving their first IVF treatment cycles.

## Materials and methods

### Study subjects

A retrospective cohort analysis was performed on the patients who underwent their first IVF treatment cycles in the Center for Reproduction Medicine of the affiliated Chenggong Hospital of Xiamen University, China, between January 2013 to December 2021. The data from cycles in the period between January 2013 to December 2018 were obtained to create models to predict total fertilization failure (development group). The data from cycles in the period between January 2019 to December 2021 were obtained to validate the models (validation group). The inclusion criteria were the patients receiving their first IVF treatments. The exclusion criteria were patients canceling their ovum pickup (OPU), patients with no oocytes, and patients with no mature oocyte at the fertilization check.

This retrospective study was approved by Institutional Review Board from the Ethical Committee of the Medical College Xiamen University. Informed consent was not necessary, because the research was based on non-identifiable records as approved by the ethics committee.

### Treatment protocol and fertilization check

Conventional agonist or antagonist stimulation protocols were used for ovarian stimulation as previously described (Cai et al., 2017). The initial and ongoing dosage was determined according to patients’ age, antral follicle count (AFC), BMI, and ovarian response. An intramuscular injection of human chorionic gonadotropin (4000–6000 IU, hCG; Livzen, China) or a subcutaneous injection of recombinant human chorionic gonadotropin (250 μg, Ovidrel, Merck-Serono, Switzerland) was administrated for final triggering when at least one follicle reached a mean diameter of 18 mm. Ovum puncture under transvaginal ultrasound guidance for oocyte retrieval was performed 34–36 h after hCG injection.

Routine IVF protocol in our center was carried out (Jiang et al., 2022). Cumulus-oocyte complexes were co-cultured with approximate 1.5–3 X 10^5^ progressively motile spermatozoa in pre-equilibrated fertilization culture medium (K-SIFM, Cook) under mineral oil in traditional incubators (C200, Labotect) at 37°C, 6% CO2 and 5% O2 in a humidified atmosphere. After 4 h co-culture, oocytes were denuded and cultured individually in preequilibrated Cleavage Medium (K-SICM, Cook). The culture system and the procedure of semen preparation were kept unchanged in the period of study. Fertilization was determined according to the presence of two pronuclei (2 PN) about 17 h post insemination. It should be confirmed 2 h later if no obvious pronuclei could be observed.

### Statistical analyses

The endpoint was total fertilization failure which was defined as the failure of all available oocytes to be fertilized in one IVF cycle. Considering the continuous variables were not normally distributed, they were presented as medians (first quartile, third quartile), while absolute frequencies and percentages (n, %) were used to present the categorical variables.

Two algorithms, a Least Absolute Shrinkage and Selection Operator (LASSO) and an Extreme Gradient Boosting (Xgboost) were used to create models. By including semen parameters or not and with only semen parameters, six different models were established in the end. Variables of female characters contained female age, duration of infertility, female primary infertility, previous IUI failure, female height, female weight, female BMI, PCOS, endometriosis, female basal FSH, female basal LH, female basal PRL, female basal E2, female basal P, female basal T, antral follicle count (AFC). Variables of male characters included male age, male primary infertility, male height, male weight, male BMI, semen volume, sperm concentration, normal morphology, sperm motility, sperm progressive motility, sperm non-progressive motility, total motile sperm count, and normozoospermia. The rest variables were the couple’s secondary infertility and ovarian stimulation characteristics, which included total gonadotropin, Gn duration, total HMG dose, HMG duration, starting dose, FSH/LH/E2/levels on the day of stimulation, total hCG dose, E2/LH/P levels on the day of triggering, follicle count over 14 mm on the day of triggering, follicle count less than 14 mm on the day of triggering, oocyte yield, count of punctured follicles, normal responder, ovarian stimulation protocol. The variables were selected according to the associations between each factor and total fertilization failure (Supplementary Figure S1 and Supplementary Figure S2). In the models of LASSO and Xgboost, three sets of models were established: 1) the fmodels with all the variables included (Lasso and Xgboost with all features), 2) the models with variables except semen parameters (Lasso and Xgboost without semen parameters), 3) the models with only semen parameters included (Lasso and Xgboost with semen parameters only).

The coefficients and intercepts of the models created with the algorithm of LASSO were shown in (Supplementary Table S1). The features showing a non-linear association with total TFF underwent a restricted cubic spline (RCS) transformation with five knots using the rms package for R software to give a better fit. Each transformed feature generates three independent spline variables following the formulas below. Each spline variable was labeled with the original feature name in combination with the numbers 1, 2, or 3. The spline variables in addition to the original feature were used to construct the models.

The receiver operating characteristic (ROC) curve with area under the curve (AUC) was calculated to qualify the predicting power of the models. A 95% confidence interval (95% CI) was calculated for the AUC. The optimal cut-off points of the ROC curves were determined according to Youden`s index. The sensitivity, specificity, positive predictive value (PPV), and negative predictive value (NPV) were calculated accordingly. The agreements between predictions and clinic observations were compared with calibration curves.

For the optimal cut-off points, the Net Reclassification index (NRI) was calculated to evaluate the contribution of semen parameters to the prediction models (Ref.). The net reclassification index (z) is defined as the difference between the correct reclassification and incorrect reclassification cases in either event (z+) or non-event (z−) patients when models were compared.

Additive NRI was defined as follows.

$$Additive\;NRI=\left(z+\right)/\left(Total\;number\;of\;patients\;with\;event\right)*100+\left(z-\right)/\left(Total\;number\;of\;patients\;without\;event\right)*100$$

Absolute NRI was defined as follows.

$$Absolute\;NRI=100*\left(\left(z+\right)+\left(z-\right)\right)/\;\left(Total\;number\;of\;patients\right)$$

Because the consequences of the prediction of the event (TFF) and non-event (fertilization) may be different. We also reported the Event-NRI and Non-Event-NRI, respectively.

$$Event-NRI=\left(z+\right)/\;\left(Total\;number\;of\;patients\;with\;an\;event\right)\;*100$$

$$Non-Event-NRI=\left(z-\right)/\;\left(Total\;number\;of\;patients\;without\;event\right)\;*100$$

The performance of the models was also evaluated in the subgroups which represent different clinical scenarios. The secondary infertility subgroup referred to the cycles of both males and females who were secondary infertile. The normozoospermia subgroup included the cycles of all parameters that met the baseline of WHO. And the normal responder subgroup included the cycles with the count of retrieved oocytes surpassing 4.

All analyses were performed using R Statistical Software (v4.1.2, R Core Team 2021).

## Result

A total of 20401 initiated cycles were identified during the study period. With the exclusion of 341 cycles cancelled for OPU, 172 cycles with no oocyte, 163 cycles resulting in no mature oocyte, and 186 cycles with miss values, 19353 cycles were included.

There were14118 cycles included in the development group, the medians of female and male age were 31.0 (28.0–34.0) and 32.0 (29.0–36.0) respectively. The duration of infertility was 3.70 (2.00–6.00). 6730 (47.7%) cycles were secondary infertility of both males and females. 12095 (85.7%) cycles were normal responders to controlled ovarian stimulation protocols and 7793 (55.2%) cycles were normozoospermia. In the end, TFF occurred in 1038 (7.4%) cycles. Another 5421 cycles were included in the validation group, the medians of female and male age were 31.0 (29.0–34.0) and 32.0 (30.0–36.0) respectively. The duration of infertility was 3.10 (2.00–5.00). 2422 (44.7%) cycles were secondary infertility of both males and females. 4638 (85.6%) cycles were normal responders and 2676 (49.4%) cycles were normozoospermia. TFF occurred in 423 (7.8%) cycles. The overall baseline characteristics of the patients were presented in Table 1.

**TABLE 1:** Cycle characteristics of patients.

	Development	Validation	Overall
	(N = 14118)	(N = 5421)	(N = 19539)
Female age,year	31.0 [28.0,34.0]	31.0 [29.0,34.0]	31.0 [28.0,34.0]
Duration of infertility, year	3.70 [2.00,6.00]	3.10 [2.00,5.00]	3.50 [2.00,5.30]
Female primary infertility	6181 (43.8%)	2418 (44.6%)	8599 (44.0%)
Previous IUI failure			
0	10734 (76.0%)	3949 (72.8%)	14683 (75.1%)
1	3384 (24.0%)	1472 (27.2%)	4856 (24.9%)
Female height,cm	158 [155,162]	158 [155,162]	158 [155,162]
Female weight,kg	53.0 [49.0,58.0]	53.0 [49.0,58.0]	53.0 [49.0,58.0]
Female BMI,kg/m^2^	21.0 [19.5,22.8]	21.3 [19.8,22.8]	21.2 [19.5,22.8]
PCOS	1034 (7.3%)	360 (6.6%)	1394 (7.1%)
Endometriosis	1629 (11.6%)	528 (9.8%)	2159 (11.1%)
Basal FSH,iu/L	6.91 [5.88,8.17]	7.28 [6.19,8.71]	7.00 [5.95,8.32]
Basal LH,iu/L	4.36 [3.22,5.84]	4.49 [3.35,6.05]	4.40 [3.26,5.89]
Basal PRL,ng/ml	13.9 [10.2,19.0]	14.3 [10.5,19.6]	14.0 [10.3,19.1]
Basal E2, pg/ml	40.0 [29.0,53.9]	42.3 [31.0,56.0]	41.0 [30.0,54.0]
Basal P,ng/ml	0.640 [0.410,0.970]	0.600 [0.390,0.890]	0.630 [0.400,0.940]
Basal T,ng/ml	0.380 [0.280,0.500]	0.450 [0.320,0.590]	0.400 [0.290,0.530]
AFC	10.0 [7.00,14.0]	10.0 [7.00,15.0]	10.0 [7.00,14.0]
**Male characteristics**			
Male age,year	32.0 [29.0,36.0]	32.0 [30.0,36.0]	32.0 [30.0,36.0]
Male primary infertility	6951 (49.2%)	2841 (52.4%)	9792 (50.1%)
Male height,cm	170 [168,174]	171 [168,175]	170 [168,175]
Male weigtht,kg	69.0 [62.0,75.0]	70.0 [63.0,77.5]	70.0 [62.0,76.0]
Male BMI,kg/m^2^	23.7 [21.5,25.9]	23.9 [21.7,26.1]	23.7 [21.5,26.0]
Semen volume,ml	2.40 [2.00,3.00]	2.40 [2.00,3.00]	2.40 [2.00,3.00]
Sperm concentration,×10^6^/ml	59.5 [36.7,91.7]	70.4 [46.0,103]	62.4 [39.1,95.2]
Normal morphology,%	6.50 [4.30,9.10]	6.00 [4.00,9.00]	6.50 [4.00,9.00]
Motility,%	48.4 [34.6,59.3]	46.0 [33.8,56.0]	47.6 [34.4,58.3]
PR,%	37.5 [25.5,47.6]	39.0 [28.3,48.8]	37.9 [26.4,48.0]
NP,%	9.50 [6.55,13.8]	5.62 [3.51,8.00]	8.23 [5.38,12.2]
TMC,×10^6^	49.9 [24.2,89.9]	62.5 [34.7,104]	53.5 [26.8,93.8]
Normal spermia	7793 (55.2%)	2676 (49.4%)	10469 (53.6%)
**ovarian stimulation parameters**			
Gonadotropin dose,iu	2250 [1800,2700]	2250 [1800,2700]	2250 [1800,2700]
Duration of stimulation,day	11.0 [10.0,13.0]	12.0 [10.0,13.0]	11.0 [10.0,13.0]
HMG dose,iu	2030 [600,2480]	1910 [600,2510]	1990 [600,2480]
HMG duration,day	11.0 [6.00,12.0]	11.0 [5.00,13.0]	11.0 [6.00,13.0]
Starting gonadotropin dose,iu	225 [150,225]	188 [150,225]	225 [150,225]
Starting day FSH,iu/L	2.48 [1.67,4.06]	2.65 [1.79,4.34]	2.53 [1.70,4.16]
Starting day LH,iu/L	0.770 [0.560,1.07]	0.750 [0.550,1.03]	0.770 [0.550,1.06]
Starting day E2,pg/ml	21.0 [12.0,32.0]	22.0 [13.0,33.0]	21.0 [13.0,32.0]
HCGdose,iu	250 [250,4000]	250 [250,250]	250 [250,4000]
Triggering day E2,pg/ml	2930 [1580,4550]	3000 [1630,4550]	2950 [1600,4550]
Triggering day LH,iu/L	0.710 [0.430,1.17]	0.670 [0.400,1.14]	0.700 [0.420,1.16]
Triggering day P,ng/ml	0.960 [0.660,1.35]	0.870 [0.610,1.20]	0.930 [0.650,1.31]
Follicles large than 14 mm	8.00 [5.00,11.0]	7.00 [5.00,10.0]	8.00 [5.00,10.0]
Follicles small than 14 mm	0 [0,1.00]	0 [0,1.00]	0 [0,1.00]
count of punctured follicles	11.0 [6.00,16.0]	10.0 [6.00,15.0]	11.0 [6.00,15.0]
Oocyte yield	9.00 [5.00,13.0]	9.00 [5.00,13.0]	9.00 [5.00,13.0]
Normal responder	12095 (85.7%)	4638 (85.6%)	16733 (85.6%)
ovarian stimulation protocol			
antagonist	2605 (18.5%)	1018 (18.8%)	3623 (18.5%)
agonist	11504 (81.5%)	4399 (81.1%)	15903 (81.4%)
others	9 (0.1%)	4 (0.1%)	13 (0.1%)
**Secondary infertility**	6730 (47.7%)	2422 (44.7%)	9152 (46.8%)
**TFF**			
no	13080 (92.6%)	4998 (92.2%)	18078 (92.5%)
yes	1038 (7.4%)	423 (7.8%)	1461 (7.5%)

**FIGURE 1:**
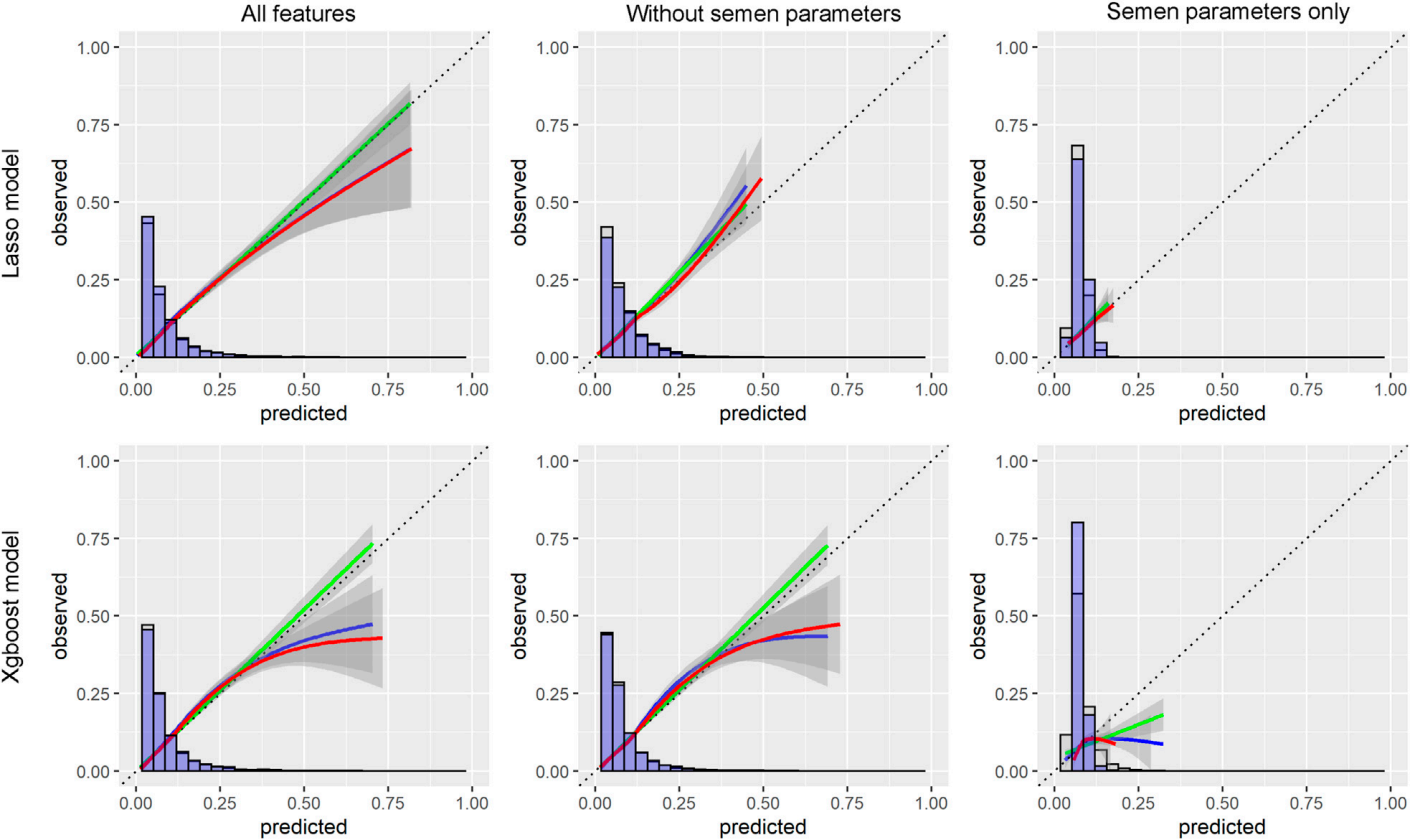
Calibration curves for predicting models.

With the LASSO algorithm, models of LASSO, LASSO without semen parameters, and LASSO with semen parameters only were established. TFF was predicted with the AUCs of .77 (95%CI:0.76-0.79), .74 (95%CI:0.73-0.76), and .58 (95%CI:0.57-0.60) respectively in development group. In the validation group, the AUCs were .74 (95%CI:0.72-0.77), .72 (95%CI:0.69-0.74), and .58 (95%CI:0.55-0.61) respectively. The other three models, Xgboost, Xgboost without semen parameters, and Xgboost with semen parameters only were created with the algorithm of Xgboost. TFF was predicted with the AUCs of .97 (95%CI:0.96-0.97), .95% (95%CI:0.95-0.96), and .85 (95%CI:0.84-0.86) respectively in development group. In the validation group, the AUCs were .75 (95%CI:0.72-0.77), .73 (95%CI:0.7-0.75), and .57 (95%CI:0.55-0.6) respectively. No matter including semen parameters or not, a comparable result could be observed from the validation group in the models of both algorithms (Table 2; Figure 1). In the models with semen parameters only, the AUCs suggested limited prediction values (Table 2, Supplementary Figure S3).

**TABLE 2:** Discrimination of different models in predicting TFF.

	LASSO	Xgboost	LASSO without semen parameters	Xgboost without semen parameters	LASSO with semen parameters only	Xgboost with semen parameters only
**Development**						
n	14100					
Prevalence (95%CI)	0.07 (0.07–0.08)					
AUC (95%CI)	0.772 (0.757,0.786)	0.966 (0.961,0.971)	0.743 (0.728,0.758)	0.953 (0.947,0.959)	0.583 (0.565,0.602)	0.853 (0.841,0.864)
cutoff	0.08	0.13	0.07	0.12	0.07	0.09
Sensitivity	0.67 (0.64,0.7)	0.89 (0.86,0.9)	0.71 (0.68,0.74)	0.86 (0.84,0.88)	0.54 (0.51,0.57)	0.73 (0.71,0.76)
Specificity	0.75 (0.75,0.76)	0.92 (0.91,0.92)	0.67 (0.66,0.67)	0.9 (0.89,0.9)	0.59 (0.58,0.6)	0.8 (0.79,0.8)
Positive predictive value	0.18 (0.17,0.19)	0.46 (0.44,0.48)	0.14 (0.13,0.15)	0.4 (0.38,0.42)	0.09 (0.09,0.1)	0.22 (0.21,0.24)
Negative predictive value	0.97 (0.96,0.97)	0.99 (0.99,0.99)	0.97 (0.96,0.97)	0.99 (0.99,0.99)	0.94 (0.94,0.95)	0.97 (0.97,0.98)
Positive likelihood ratio	2.73 (2.59,2.87)	10.7 (10.11,11.42)	2.12 (2.03,2.23)	8.38 (7.93,8.86)	1.31 (1.24,1.39)	3.6 (3.43,3.78)
Negative likelihood ratio	0.44 (0.4,0.48)	0.13 (0.11,0.15)	0.43 (0.39,0.48)	0.15 (0.13,0.18)	0.78 (0.73,0.83)	0.33 (0.3,0.37)
Diagnostic odds ratio	6.24 (5.44,7.17)	85.9 (70.22,106.11)	4.89 (4.25,5.64)	54.9 (45.55,66.66)	1.69 (1.48,1.92)	10.8 (9.32,12.5)
Accuracy	0.75 (0.74,0.76)	0.92 (0.91,0.92)	0.67 (0.66,0.68)	0.9 (0.89,0.9)	0.58 (0.58,0.59)	0.79 (0.78,0.8)
						
**Validation**						
n	5421					
Prevalence (95%CI)	0.08 (0.07–0.09)					
AUC (95%CI)	0.74 (0.72,0.77)	0.75 (0.72,0.77)	0.72 (0.69,0.74)	0.73 (0.7,0.75)	0.58 (0.55,0.61)	0.57 (0.55,0.6)
cutoff	0.08	0.13	0.07	0.12	0.07	0.09
Sensitivity	0.63 (0.59,0.68)	0.41 (0.36,0.45)	0.69 (0.64,0.73)	0.41 (0.37,0.46)	0.54 (0.5,0.59)	0.34 (0.3,0.39)
Specificity	0.74 (0.73,0.75)	0.88 (0.87,0.89)	0.64 (0.62,0.65)	0.87 (0.86,0.88)	0.59 (0.57,0.6)	0.75 (0.74,0.77)
Positive predictive value	0.17 (0.15,0.19)	0.22 (0.19,0.25)	0.14 (0.12,0.15)	0.22 (0.19,0.24)	0.1 (0.09,0.11)	0.11 (0.09,0.12)
Negative predictive value	0.96 (0.95,0.97)	0.95 (0.94,0.95)	0.96 (0.95,0.97)	0.95 (0.94,0.95)	0.94 (0.93,0.95)	0.93 (0.92,0.94)
Positive likelihood ratio	2.45 (2.26,2.66)	3.35 (3.01,3.74)	1.89 (1.75,2.04)	3.24 (2.91,3.61)	1.32 (1.2,1.44)	1.39 (1.26,1.54)
Negative likelihood ratio	0.49 (0.44,0.56)	0.68 (0.62,0.73)	0.49 (0.43,0.57)	0.67 (0.62,0.73)	0.78 (0.7,0.87)	0.87 (0.81,0.94)
Diagnostic odds ratio	4.96 (4.01,6.15)	4.96 (3.99,6.17)	3.84 (3.09,4.79)	4.82 (3.88,5.98)	1.69 (1.38,2.08)	1.59 (1.28,1.98)
Accuracy	0.73 (0.72,0.74)	0.84 (0.83,0.85)	0.64 (0.63,0.65)	0.84 (0.83,0.85)	0.58 (0.57,0.6)	0.72 (0.71,0.73)

All features, models with all the variables were included; without semen parameters, models with variables except semen parameters were included; models including only semen parameters.

The importance of each variable in different models was computed with the Xgboost algorithm. The oocyte yield was the most important variable in predicting the occurrence of TFF no matter in the model with or without semen parameters. In the model of semen parameters only, TMC, motility, and sperm concentration showed the best importance (Supplementary Figure S4).

The prediction of TFF was stratified according to the patient subgroup. The TFF occurrence rates ranged from 6% to 21% in different subgroups. A relatively lower prevalence of 6% was observed in the group of secondary infertility, normozoospermia, and normal responder. The prevalence increased in the rest groups, and the highest was achieved in the poor responder group at 21%. The models with or without semen parameters had comparable predictive values and were better than the models with semen parameters only according to the AUCs (Table 3, Supplementary Figure S5).

**TABLE 3:** Discrimination of different models in predicting TFF in different patient subgroup.

	Secondary infertility	Primary infertility	Normozoospermia	Male subfertility	Normal responder	Poor responder
Sample size	2420	3000	2680	2740	4640	783
**Prevalence (95% CI)**	0.06 (0.05,0.07)	0.09 (0.08,0.11)	0.06 (0.05,0.07)	0.09 (0.08,0.1)	0.06 (0.05,0.06)	0.21 (0.18,0.24)
**AUC (95% CI)**						
LASSO	0.75 (0.71,0.8)	0.73 (0.7,0.76)	0.75 (0.71,0.79)	0.73 (0.7,0.76)	0.7 (0.66,0.73)	0.66 (0.61,0.7)
Xgboost	0.76 (0.72,0.8)	0.72 (0.69,0.75)	0.75 (0.71,0.79)	0.73 (0.7,0.76)	0.69 (0.66,0.72)	0.69 (0.65,0.74)
LASSO without semen parameters	0.72 (0.68,0.77)	0.7 (0.66,0.73)	0.74 (0.71,0.78)	0.7 (0.66,0.73)	0.67 (0.63,0.7)	0.61 (0.56,0.66)
Xgboost without semen parameters	0.73 (0.69,0.78)	0.7 (0.67,0.73)	0.76 (0.72,0.79)	0.7 (0.67,0.74)	0.66 (0.63,0.69)	0.69 (0.65,0.74)
LASSO with semen parameters only	0.58 (0.52,0.63)	0.58 (0.54,0.61)	0.52 (0.48,0.57)	0.57 (0.54,0.61)	0.61 (0.57,0.64)	0.53 (0.48,0.58)
Xgboost with semen parameters only	0.56 (0.51,0.6)	0.58 (0.55,0.62)	0.53 (0.49,0.58)	0.57 (0.53,0.6)	0.59 (0.55,0.62)	0.54 (0.49,0.59)
**Diagnostic odds ratio (95% CI)**						
LASSO	5.65 (3.91,8.15)	4.45 (3.38,5.92)	5.2 (3.73,7.27)	4.54 (3.42,6.07)	3.67 (2.82,4.78)	3.2 (1.81,6.05)
Xgboost	7.06 (4.69,10.56)	3.96 (3.04,5.16)	6.14 (4.26,8.8)	4.12 (3.12,5.44)	3.19 (2.26,4.44)	3.17 (2.14,4.76)
LASSO without semen parameters	4.46 (3.12,6.41)	3.25 (2.44,4.39)	4.59 (3.22,6.62)	3.37 (2.55,4.48)	2.7 (2.08,3.51)	1.79 (0.82,4.45)
Xgboost without semen parameters	7.26 (4.79,10.89)	3.79 (2.91,4.93)	6.03 (4.28,8.47)	4.07 (3.05,5.41)	2.96 (2.1,4.12)	3.27 (2.19,4.95)
LASSO with semen parameters only	1.5 (1.05,2.14)	1.78 (1.38,2.3)	1.15 (0.71,1.8)	1.78 (1.29,2.51)	2 (1.54,2.6)	1.26 (0.88,1.8)
Xgboost with semen parameters only	1.2 (0.8,1.76)	1.83 (1.4,2.38)	1.01 (0.57,1.71)	1.55 (1.19,2.03)	1.75 (1.33,2.29)	1.33 (0.89,1.96)
**Accuracy (95% CI)**						
LASSO	0.84 (0.83,0.85)	0.65 (0.63,0.66)	0.79 (0.77,0.8)	0.68 (0.66,0.7)	0.79 (0.78,0.8)	0.38 (0.35,0.42)
Xgboost	0.9 (0.89,0.91)	0.8 (0.78,0.81)	0.88 (0.87,0.9)	0.8 (0.79,0.82)	0.89 (0.88,0.9)	0.58 (0.54,0.61)
LASSO without semen parameters	0.78 (0.76,0.79)	0.53 (0.52,0.55)	0.65 (0.63,0.67)	0.63 (0.61,0.65)	0.71 (0.69,0.72)	0.27 (0.24,0.3)
Xgboost without semen parameters	0.9 (0.89,0.91)	0.78 (0.77,0.8)	0.85 (0.84,0.87)	0.82 (0.81,0.84)	0.88 (0.87,0.89)	0.57 (0.54,0.6)
LASSO with semen parameters only	0.59 (0.57,0.61)	0.58 (0.56,0.6)	0.82 (0.81,0.84)	0.35 (0.33,0.37)	0.59 (0.57,0.6)	0.56 (0.52,0.59)
Xgboost with semen parameters only	0.73 (0.71,0.74)	0.72 (0.7,0.73)	0.85 (0.84,0.86)	0.6 (0.58,0.62)	0.73 (0.72,0.75)	0.66 (0.62,0.69)

All features, models with all the variables were included; without semen parameters, models with variables except semen parameters were included; models including only semen parameters.

To improve the performance of the models in the poor responder group, we update the models, which were calibrated according to the slope and intercept of the calibration curve. At the same time, we created refitted models, which were constructed from the development data with poor responders only. The coefficients of LASSO models refitted for poor responders were presented in (Supplementary Table S2). The models provided moderate performance after being updated and refitted according to AUCs and calibration curves (Supplementary Table S3 and Supplementary Figure S6).

The NRIs for the overall validation cohort and subgroups suggested that including semen parameters may have a positive effect on the classification of the patients as it provided positive NIR values (Supplementary Table S4). However, the event NRI values suggested that adding semen parameters to the models harms the correct classification of patients with TFF while the positive non-event NRI indicated that it contributed to the classification of patients without TFF. Nevertheless, in the models refitted for poor responders, semen parameters added little or none to absolute NRI and non-event NRI.

## Discussion

### Main finding

In the present study, we used two algorithms, a Least Absolute Shrinkage and Selection Operator (LASSO) and an Extreme Gradient Boosting (Xgboost), to develop predictive models for TFF in a retrospective cohort including 19539 cycles receiving their first IVF treatments in our center from January 2013 to December 2021. It was found that including semen parameters would limitedly improve the prediction power of the models for TFF in the cycles of IVF. The models without semen parameters could achieve comparable predictive power with the models with semen parameters according to AUCs. On the other hand, the models with only semen parameters had nearly no meaningful predicting value. The count of retrieved oocytes was the most important factor associated with the occurrence of TFF. Furthermore, in the cycles of poor responders, we observed a higher prevalence of TFF and our models could give reasonable predictions.

### Interpretation

With or without semen parameters, our models yield comparable discriminatory powers with previous studies (Repping et al., 2002; Li J. et al., 2021; Tian et al., 2022). However, the heterogeneity in the inclusion criteria, outcomes measured, and study design might hamper further comparison between our models and previous ones. For instance, the models of Tian et al. (2022) reported an AUC for TFF in both IVF and ICSI cycles, and the work of Li J. et al. (2021) aimed to predict the combined incidence of TFF and low fertilization rate. Importantly, several studies were not limited to the first cycle (Repping et al., 2002; Li J. et al., 2021; Tian et al., 2022). As ICSI could be used to overcome the failure of fertilization in previous cycles, including multiple cycles for model development risks a potential selection bias. Also, our study was the only study including a temporary validation following model development among the studies mentioned (Repping et al., 2002; Li J. et al., 2021; Tian et al., 2022). The validation warned of a risk of overfitting in certain algorithms. With the same predictors, the Xgboost models underwent a dramatic decrease in discriminatory power while the discriminatory power of the LASSO models remained stable. Because a minimal standard deviation of the residuals has been secured in the internal validation during the development of Xgboost, our data also suggested a risk of over-fit in models that lack external validation.

Our findings appear to conflict with the work of Repping et al. (2002) with semen parameters alone, we failed to demonstrate a meaningful discriminatory power of the models. It could be explained by the difference in the criteria of ICSI. The study of Repping et al. (2002) used a criterion of .2 million post-wash progressive sperm for ICSI, which was much lower than ours. Their cohort may include more patients with poor semen parameters and thus with more TFF cases due to insufficient sperm input. Supporting the hypothesis, their data also demonstrated a TFF rate (110/892) higher than both our study and the Vienna criteria (ESHRE Special Interest Group of Embryology and Alpha Scientists in Reproductive Medicine, 2017).

A further question is whether a prediction model is suitable for different clinical scenarios. While most of the previous models were based on unselected IVF patients (Repping et al., 2002; Li J. et al., 2021; Tian et al., 2022), the work of Li J. et al. (2021) is based on patients with boarder line semen parameters. The fact that the selection of insemination protocol depends on the semen quality (Dcunha et al., 2022) might lead to bias with respect to the difference between the population in which the predictive models were developed and the patients who are supposed to have high risks. A one-shoe-for-all model developed in an unselected population might not meet all potential clinical scenarios. In our subgroup analyses, TFF occurred with a prevalence of 6% and 9% in the normozoospermia patients and the patients with sub-optimal semen parameters. Nevertheless, the discriminatory power and the calibration in the large remained similar between the two subgroups, suggesting a similar overall performance of the model.

Besides the clinical characteristics of the patients, TFF may also result from genetic deficiencies (Koler et al., 2009; Litzky and Marsit, 2019; Jin et al., 2021; Li M. et al., 2021; Liu et al., 2021; Wang et al., 2021), which are not necessarily related to the clinical predictors such as semen parameters. It could be argued that a clinical prediction is useless if a significant part of the TFF patients suffered from undetected genetic deficiencies. However, the extent of TFF patients who are affected by genetic deficiencies is largely unknown. The diagnosis of secondary fertility suggests at least one previous successful fertilization that occurred *in vivo* in those patients. Therefore, patients with secondary infertility are supposed to be unaffected by the genetic deficiencies which impair the fertilization process. With similar AUCs demonstrated in both primary and secondary infertility patients, it might suggest absolute deficiencies that affect fertilization are rare events even for TFF patients. It also echoes the earlier studies which suggested that patients suffering from TFF might have an increased fertilization rate in the second cycle when conventional IVF continues.

Previous studies had demonstrated a significant association between oocyte yield and TFF (Sarikaya et al., 2011; Xia et al., 2020; Tian et al., 2022). By adding the oocyte yield to the model, Repping et al. (2002) increased the discriminatory power of their model to .8. In the present study, the Xgboost model suggested that the oocyte yield was the predictor of the highest importance. The prevalence of TFF in poor responders was as high as .21(95%CI:0.18-0.24). These data highlighted the role of the female factor in the occurrence of TFF. Although one might argue that fertilization still manifested a probability event for an individual oocyte and TFF may occur in poor responders just by chance, the models refitted for poor responders showed a moderate discriminatory power. It may encourage future studies focusing on the prediction of TFF for poor responders.

### Clinical significance

In practice, TFF threatened a small fraction of infertility patients. The reported prevalence was 5%–20% in previous studies (Combelles et al., 2010; Huang et al., 2015). In the present study, we observed a less than 8% prevalence in the whole IVF treatment cycles of our center. By naïve guessing that all cycles were fertilized, an accuracy higher than 90% will be achieved. In these terms of view, it appeared that predicting TFF has limited benefits. However, the high prevalence of TFF in poor responders is unneglectable. As the evidence does not support the use of ICSI for all poor responders (6), it is worth the efforts to predict the poor responders with higher risks for TFF.

While a predictive model provides a positive or negative prediction for individual patients, the consequences of having a positive prediction may not always be the same as those of having a negative prediction. In the case of TFF, if the clinical decision were made according to the prediction, the patients misclassified as TFF may be incorrectly assigned with ICSI, increasing the potential treatment burden, but the treatment continues. On the other hand, for the patients misclassified as “healthy”, the cycle is canceled. If the goal of a prediction model is to prevent the latter situation, discriminability or accuracy might not be the only target to purchase. According to our data, a hypothetical poor responder cohort including 100 IVF patients may have 20 cases of unexpected TFF. With conventional IVF for all patients, 20 cycles may be cancelled. If the decision was made according to the model, approximately 8 TFF patients were misclassified as “healthy” and thus canceled the cycle, according to the average sensitivity of the model, while 16“healthy” patients received ICSI. With an ICSI for all policy, the ICSI cycles for “healthy” patients increase to 80.

### Strengths and limitations

Only cycles of the first IVF treatment were included in our study, the results were more practicable for insemination protocol decision-making, and the prediction power was ensured by a large sample size. We also calibrate the models in a different data set and different clinic scenarios, such as normozoospermia or male subfertility, normal or poor responder, and primary or secondary infertility. Some drawbacks must be concerned, such as retrospective analysis study design, and data set from a single IVF center. So, it is necessary to test the performance of the models in more IVF centers with different treatment systems. The study also failed to include certain clinical parameters, such as DNA fragmentation and male endocrine parameters, which may further improve the discriminatory power of the models.

## Conclusion

Our study showed that including basic semen parameters would limitedly improve the prediction value for the occurrence of total fertilization failure in the first IVF cycles. On the other hand, the oocyte yields were significantly associated with the TFF and the prevalence of TFF in poor responders was high. With our models, a reasonable predicting value could be achieved, especially practicable in the poor responder treatment cycles.

## Data Availability

The original contributions presented in the study are included in the article/Supplementary Material, further inquiries can be directed to the corresponding author.
