# Supplementary material for: Predicting the unexpected total fertilization failure in conventional in vitro fertilization cycles: What is the role of semen quality?
Source: Front Cell Dev Biol. 2023 Feb 23;11:1133512. doi: 10.3389/fcell.2023.1133512 (PMC9996289; doi:10.3389/fcell.2023.1133512)
Supplement: Supplementary file 1 [file Table1.DOCX]

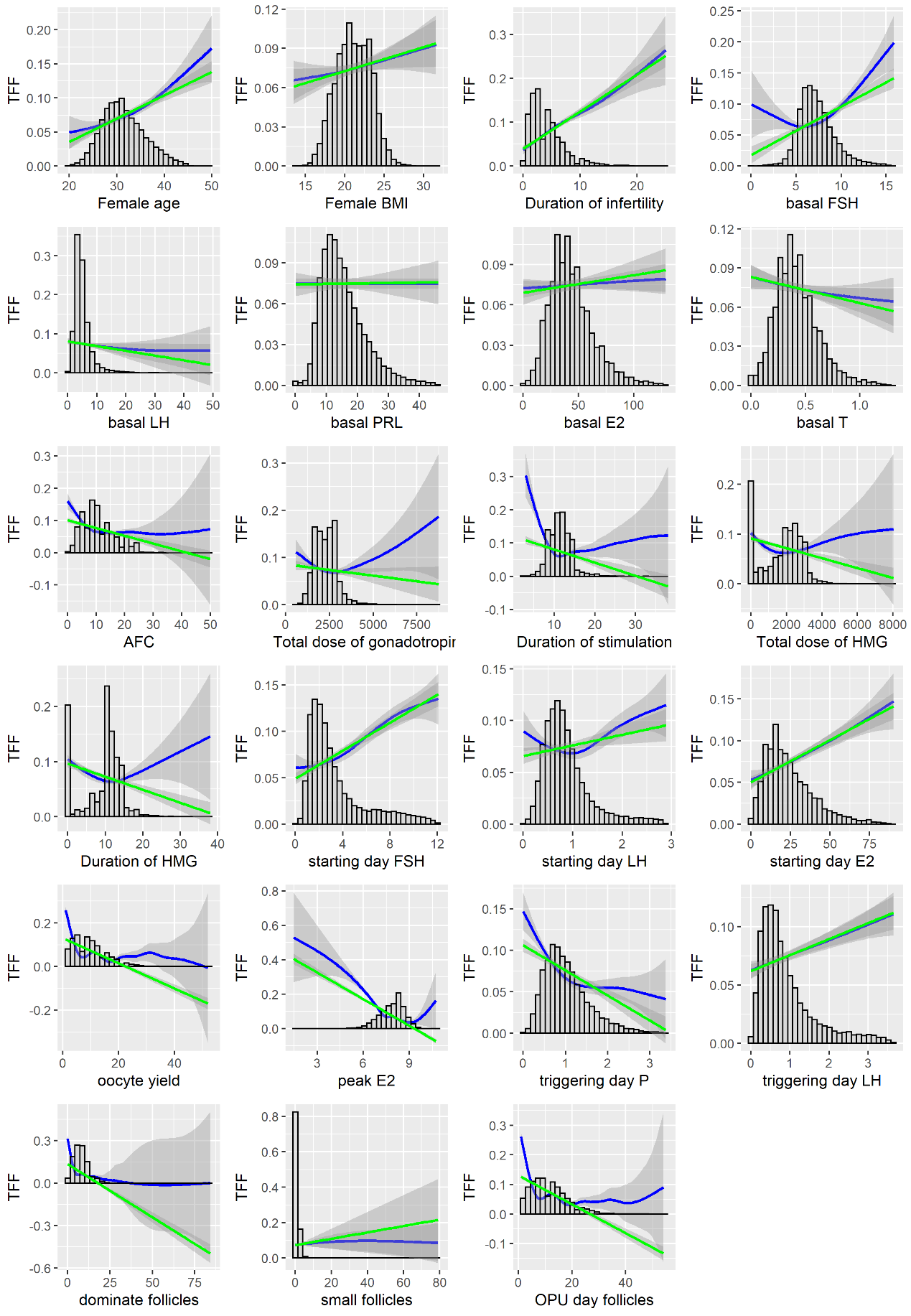


Figure S1 Association between female factors and total fertilization failure (TFF). The green line indicates a linear fit. The blue line indicates a smooth fit. The shades indicate confident intervals. The histograms were added to indicate sparse data regions. Dominate follicles, follicles ≧14mm. Small follicles, follicles < 14mm.


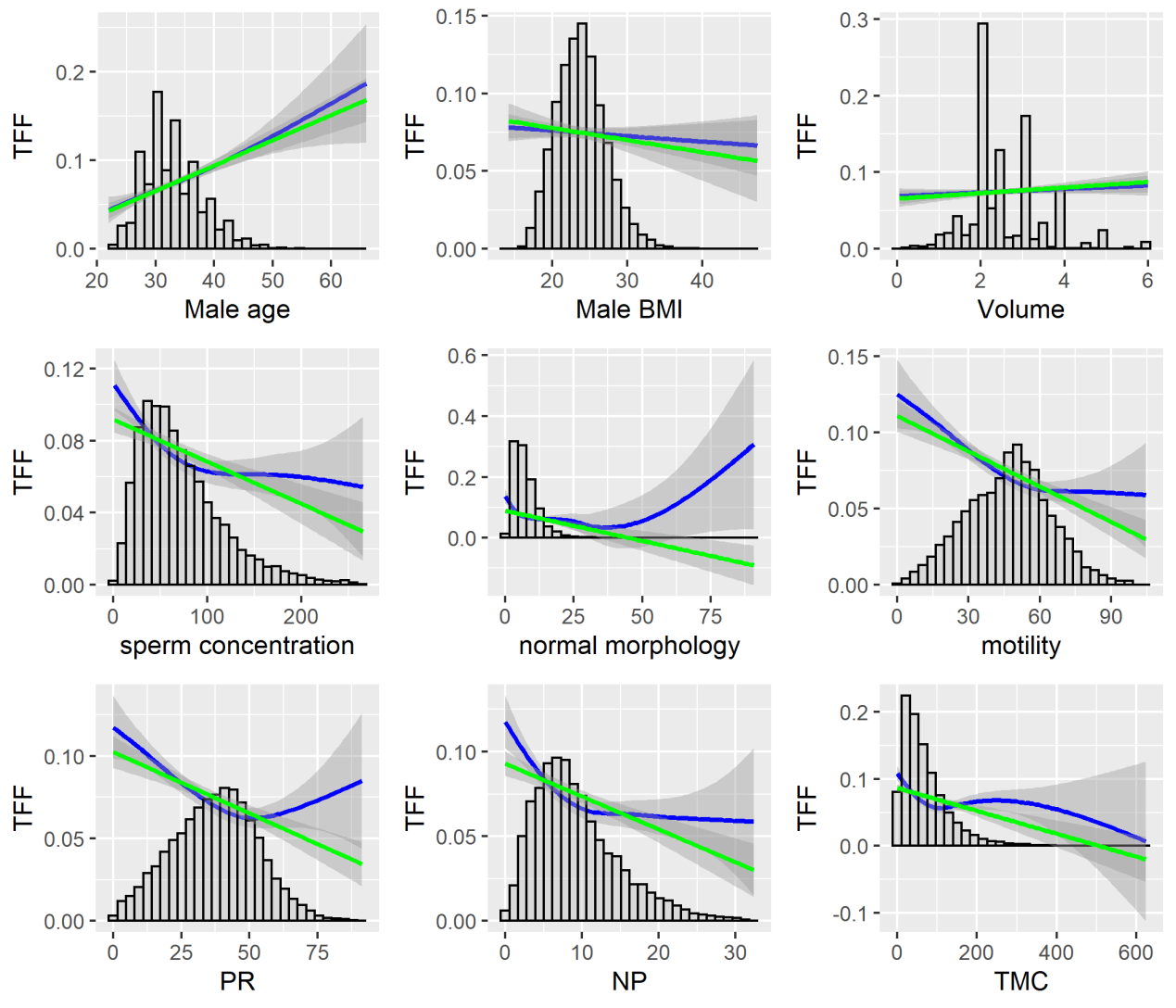


Figure S2 Association between male factors and total fertilization failure (TFF). The green line indicates a linear fit. The blue line indicates a smooth fit. The shades indicate confident intervals. The histograms were added to indicate sparse data regions. PR, progressive motility (%); NP, non-progressive motility (%); TMC, total motile sperm count (million).


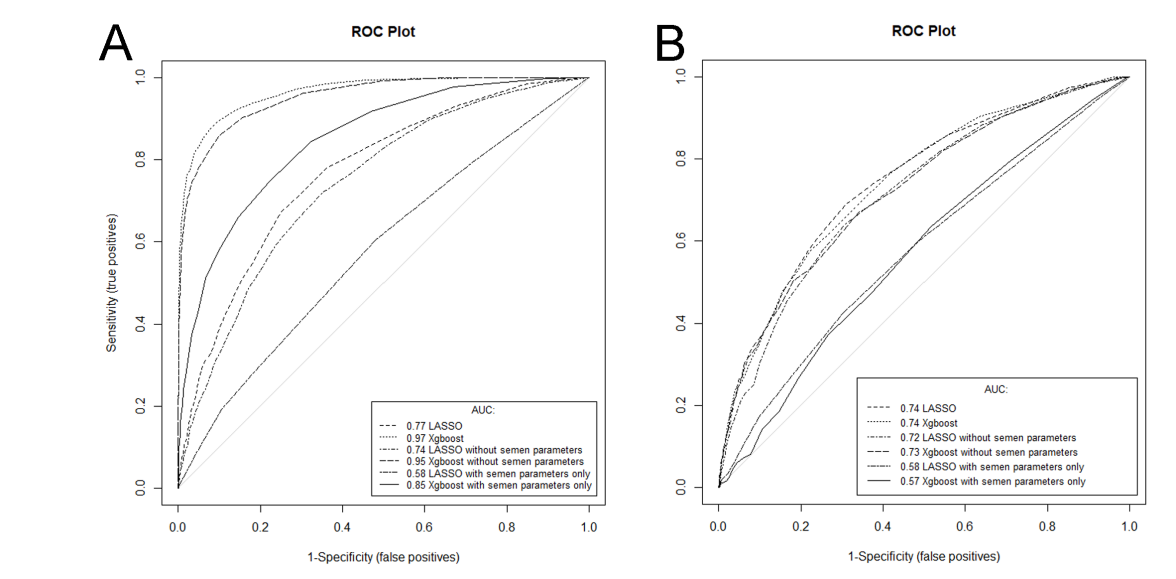


Figure S3 ROC curves of predicting models in developmental (A) and validation (B) data. All features, models with all the variables were included; without semen parameters, models with variables except semen parameters were included; models including only semen parameters


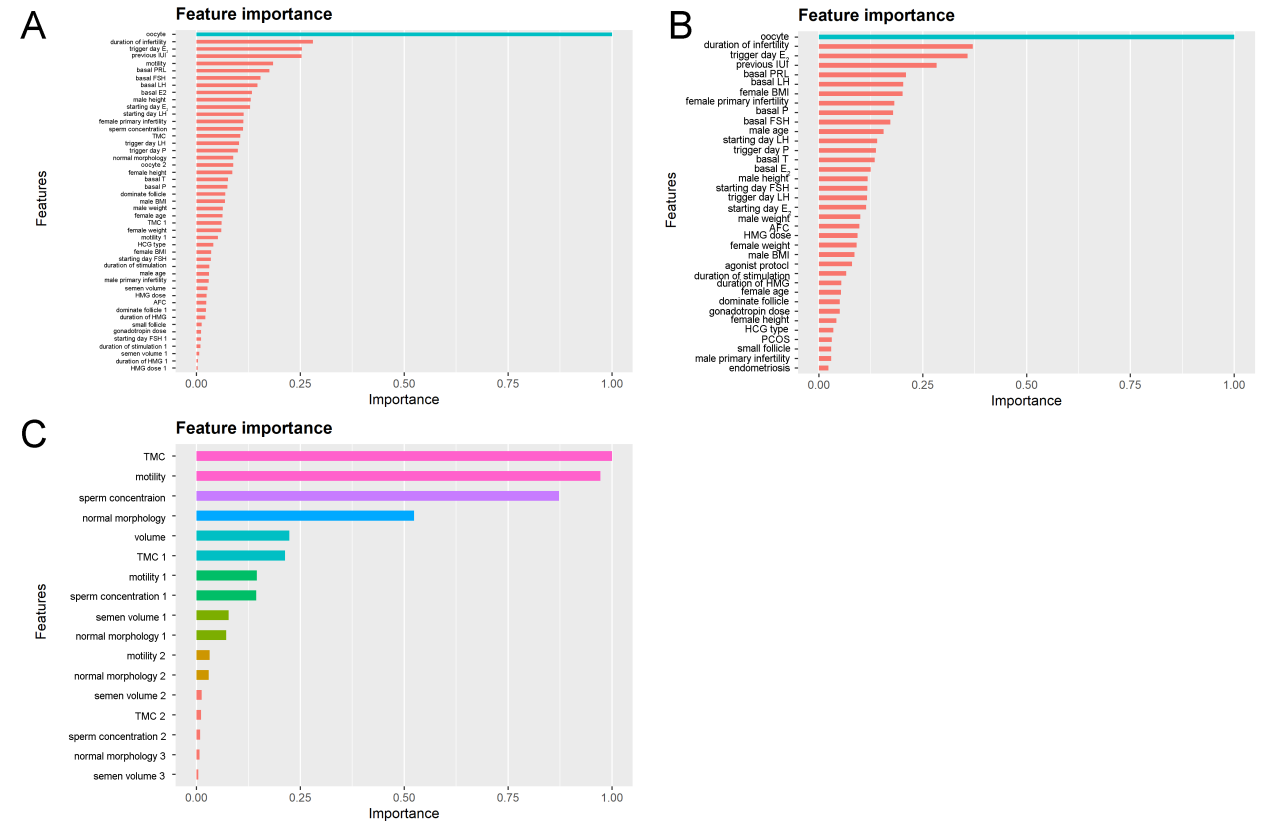


Figure S4 Important features of Xgboost models using full set features (A), features without semen parameters (B), and features with semen parameters only (C).


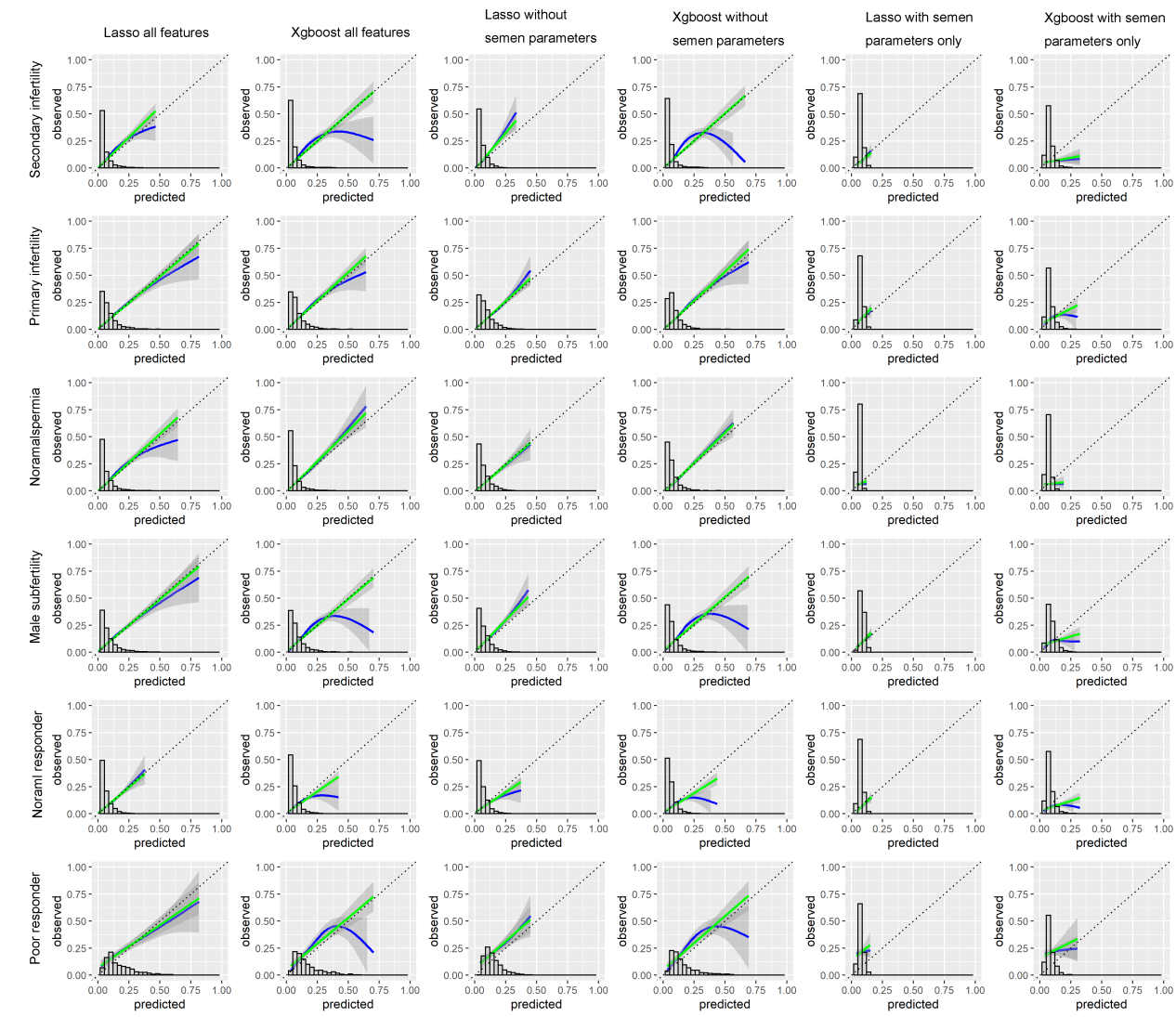


Figure S5 Calibration curves for subgroups in validation data. The predicted chance of total fertilization failure was plotted against the observed incidence. The green line indicates a linear fit. The blue line indicates a smooth fit. The dotted line indicates a perfect fit. The shades indicate confident intervals. The histograms were added to indicate sparse data regions.All features, models with all the variables were included; without semen parameters, models with variables except semen parameters were included; models including only semen parameters


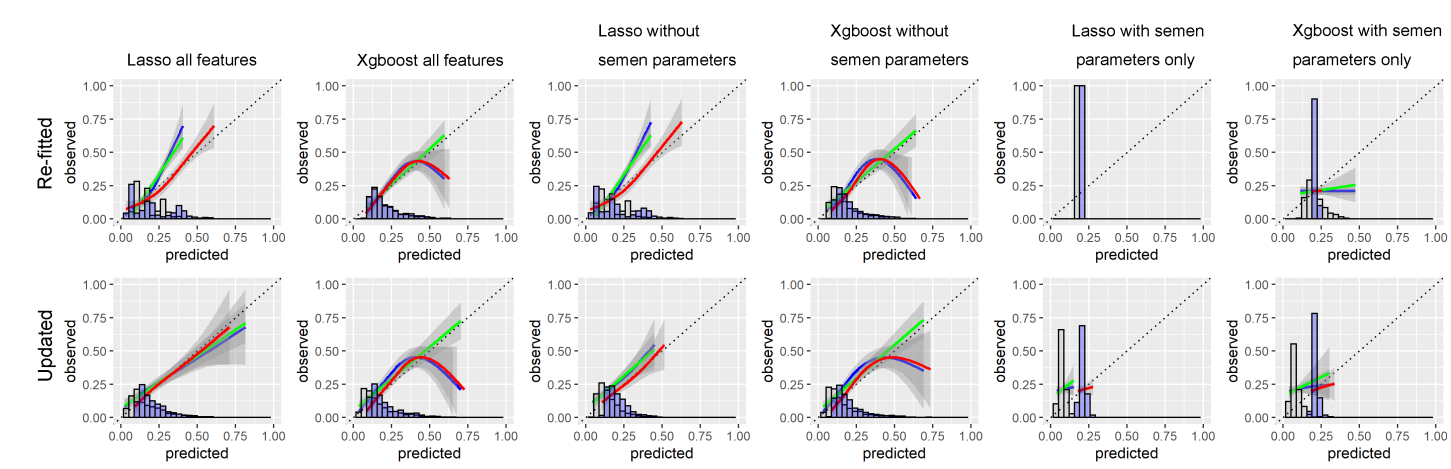


Figure S6 Calibration curves for poor responders in validation data. The predicted chance of total fertilization failure was plotted against the observed incidence in the re-fitted model and the updated model. The green line indicates a linear fit. The blue line indicates a smooth fit. The red line indicates a smooth fit rescaled according to the slope and intercept of the linear fit. The dotted line indicates a perfect fit. The shades indicate confident intervals. The grey histograms indicate sparse data regions for models before rescaling. The blue histograms indicate sparse data regions for models after rescaling.All features, models with all the variables were included; without semen parameters, models with variables except semen parameters were included; models including only semen parameters

Table S1 Coefficients of Lasso models

|  | Lasso all features | Lasso without semen | Lasso semen only |
| --- | --- | --- | --- |
| Intercept | 19.460190 | -1.765849 | -1.537029 |
| Female age | -0.037043 | -0.019138 | N/A |
| Mae age | 0.032220 | 0.024838 | N/A |
| Duration of infertility | 0.076634 | 0.068054 | N/A |
| Female primary infertility | 0.419155 | 0.406699 | N/A |
| Male primary infertility | 0.223742 | 0.220827 | N/A |
| PCOS | -0.739815 | -0.621434 | N/A |
| Endometriosis | 0.039895 | . | N/A |
| Previous IUI attempt | 0.803814 | 0.739123 | N/A |
| Female height | -0.081212 | -0.003147 | N/A |
| Female weight | 0.106166 | . | N/A |
| Female BMI | -0.271271 | 0.001550 | N/A |
| Male height | -0.040397 | . | N/A |
| Male weight | 0.039855 | -0.004619 | N/A |
| Male BMI | -0.130044 | . | N/A |
| Semen volume | 0.384683 | N/A | 0.050453 |
| Semen volume1 | -1.178958 | N/A | -0.002424 |
| Semen volume2 | 2.833638 | N/A | . |
| Semen volume3 | -0.917149 | N/A | . |
| sperm concentration | -0.005834 | N/A | -0.003772 |
| sperm concentration1 | 0.014592 | N/A | 0.002424 |
| sperm concentration2 | . | N/A | 0.000414 |
| sperm concentration3 | -0.040818 | N/A | . |
| sperm normal morphology | -0.143928 | N/A | -0.070055 |
| sperm normal morphology1 | 0.877730 | N/A | 0.060712 |
| sperm normal morphology2 | -1.674115 | N/A | . |
| sperm normal morphology3 | 0.791480 | N/A | . |
| sperm motility | -0.007189 | N/A | -0.010077 |
| sperm motility1 | -0.006657 | N/A | . |
| sperm motility2 | 0.025269 | N/A | . |
| sperm motility3 | . | N/A | 0.025905 |
| TMC | -0.005341 | N/A | -0.002120 |
| TMC 1 | 0.012922 | N/A | 0.001526 |
| TMC 2 | . | N/A | 0.001713 |
| TMC 3 | -0.023926 | N/A | 0.001336 |
| Basal FSH | 0.021374 | 0.018615 | N/A |
| Basal LH | -0.017852 | -0.010052 | N/A |
| Basal PRL | 0.001209 | . | N/A |
| Basal E2 | 0.000857 | 0.000132 | N/A |
| Basal P | 0.018899 | 0.017443 | N/A |
| Basal T | 0.195379 | 0.109133 | N/A |
| AFC | 0.026033 | 0.016118 | N/A |
| Agonist | 0.713821 | . | N/A |
| nonAgonist | 0.772779 | N/A | N/A |
| gonadotropin dose | 0.000315 | -0.000068 | N/A |
| gonadotropin dose1 | -0.000403 | . | N/A |
| gonadotropin dose2 | . | . | N/A |
| gonadotropin dose3 | 0.000883 | -0.000202 | N/A |
| duration of stimulation | -0.166967 | -0.002932 | N/A |
| duration of stimulation1 | 0.420726 | . | N/A |
| duration of stimulation2 | -1.491460 | . | N/A |
| duration of stimulation3 | 0.751984 | . | N/A |
| HMG dose | 0.000146 | . | N/A |
| HMG dose1 | -0.000056 | . | N/A |
| HMG dose2 | . | . | N/A |
| HMG dose3 | -0.000933 | . | N/A |
| HMG duration | 0.039688 | 0.003331 | N/A |
| HMG duration1 | -0.158817 | . | N/A |
| HMG duration2 | 1.220232 | 0.040818 | N/A |
| HMG duration3 | -4.645468 | . | N/A |
| Starting dose | 0.000365 | . | N/A |
| starting day FSH | 0.027775 | . | N/A |
| starting day FSH1 | 0.112654 | . | N/A |
| starting day FSH2 | . | . | N/A |
| starting day FSH3 | -0.468272 | . | N/A |
| starting day LH | 0.003333 | . | N/A |
| starting day E2 | 0.006575 | 0.005913 | N/A |
| HCG type | 0.000002 | 0.000005 | N/A |
| triggering day E2 | -0.000032 | -0.000066 | N/A |
| triggering day E2 1 | 0.000229 | . | N/A |
| triggering day E2 2 | -0.001889 | . | N/A |
| triggering day E2 3 | 0.003687 | 0.000245 | N/A |
| triggering day LH | 0.002856 | -0.013108 | N/A |
| triggering day P | -0.552421 | -0.050218 | N/A |
| triggering day P 1 | 1.403282 | . | N/A |
| triggering day P 2 | 0.551786 | . | N/A |
| triggering day P 3 | -5.660928 | 0.066980 | N/A |
| dominate follicle | -0.071874 | -0.002804 | N/A |
| dominate follicle ≧14 mm 1 | 0.740681 | . | N/A |
| dominate follicle ≧14 mm 2 | -1.887283 | . | N/A |
| dominate follicle ≧14 mm 3 | 1.426143 | . | N/A |
| small follicle | 0.019905 | . | N/A |
| Oocyte yield | -0.543093 | -0.175708 | N/A |
| Oocyte yield 1 | 4.285050 | 0.135302 | N/A |
| Oocyte yield 2 | -9.166727 | . | N/A |
| Oocyte yield 3 | 7.855703 | . | N/A |

“.” indicates the coefficient is reduced in the Lasso model.

“N/A” indicates the feature is not applicable in the model.

The features showing a non-linear association with total fertilization failure (TFF) underwent a restricted cubic spline (RCS) transformation with 5 knots using the rms package for R software to give a better fit. Each transformed feature generates 3 independent spline variables following the formulas below. Each spline variable was labeled with the original feature name in combination with the numbers 1, 2, or 3. The spline variables in addition to the original feature were used to construct the models.

**RCS transformation formulas**

semen volume 1=（MAX(semen volume-**1.3**,0)^3-((4-**1.3**)*(MAX(semen volume-3,0)^3)+(3-**1.3**)*(MAX(semen volume-4,0)^3))/(4-3))/7.29

semen volume 2=（MAX(semen volume-**2**,0)^3-((4-**2**)*(MAX(semen volume-3,0)^3)+(3-**2**)*(MAX(semen volume-4,0)^3))/(4-3))/7.29

semen volume 3=（MAX(semen volume-**2.4**,0)^3-((4-**2.4**)*(MAX(semen volume-3,0)^3)+(3-**2.4**)*(MAX(semen volume-4,0)^3))/(4-3))/7.29

sperm concentration 1=（MAX(sperm concentration-**18.6**,0)^3-((164.1-**18.6**)*(MAX(sperm concentration-91.05,0)^3)+(91.05-**18.6**)*(MAX(sperm concentration-164.1,0)^3))/(164.1-91.05))/21170.25

sperm concentration 2=（MAX(sperm concentration-**41.3**,0)^3-((164.1-**41.3**)*(MAX(sperm concentration-91.05,0)^3)+(91.05-**41.3**)*(MAX(sperm concentration-164.1,0)^3))/(164.1-91.05))/21170.25

sperm concentration 3=（MAX(sperm concentration-6**2.4**,0)^3-((164.1-**62.4**)*(MAX(sperm concentration-91.05,0)^3)+(91.05-**62.4**)*(MAX(sperm concentration-164.1,0)^3))/(164.1-91.05))/21170.25

normal morphology 1=（MAX(normal morphology-**2.5**,0)^3-((16-**2.5**)*(MAX(normal morphology-9,0)^3)+(9-**2.5**)*(MAX(normal morphology-16,0)^3))/(16-9))/182.25

normal morphology 2=（MAX(normal morphology-**4.5**,0)^3-((16-**4.5**)*(MAX(normal morphology-9,0)^3)+(9-**4.5**)*(MAX(normal morphology-16,0)^3))/(16-9))/182.25

normal morphology 3=（MAX(normal morphology-**6.5**,0)^3-((16-**6.5**)*(MAX(normal morphology-9,0)^3)+(9-**6.5**)*(MAX(normal morphology-16,0)^3))/(16-9))/182.25

motility1=（MAX(motility-**16.949**,0)^3-((75-**16.949**)*(MAX(motility-57.1,0)^3)+(57.1-**16.949**)*(MAX(motility-75,0)^3))/(75-57.1))/3359

motility2=（MAX(motility-**35.96**,0)^3-((75-**35.96**)*(MAX(motility-57.1,0)^3)+(57.1-**35.96**)*(MAX(motility-75,0)^3))/(75-57.1))/3359

motility3=（MAX(motility-**47.64**,0)^3-((75-**47.64**)*(MAX(motility-57.1,0)^3)+(57.1-**47.64**)*(MAX(motility-75,0)^3))/(75-57.1))/3359

TMC 1=（MAX(TMC-**7.186497**,0)^3-((189.4258-**7.186497**)*(MAX(TMC-88.46236,0)^3)+( 88.46236-**7.186497**)*(MAX(TMC-189.4258,0)^3))/( 189.4258- 88.46236))/33211.16

TMC 2=（MAX(TMC- **29.135584**,0)^3-((189.4258-**29.135584**)*(MAX(TMC-88.46236,0)^3)+( 88.46236-**29.135584**)*(MAX(TMC-189.4258,0)^3))/( 189.4258- 88.46236))/33211.16

TMC 3=（MAX(TMC- **53.464537**,0)^3-((189.4258-**53.464537**)*(MAX(TMC-88.46236,0)^3)+( 88.46236-**53.464537**)*(MAX(TMC-189.4258,0)^3))/( 189.4258- 88.46236))/33211.16

gonadotropin dose 1=（MAX(gonadotropin dose-**1350**,0)^3-((3375-**1350**)*(MAX(gonadotropin dose-2700,0)^3)+(2700-**1350**)*(MAX(gonadotropin dose-3375,0)^3))/(3375-2700))/4100625

gonadotropin dose 2=（MAX(gonadotropin dose- **1875**,0)^3-((3375- **1875**)*(MAX(gonadotropin dose-2700,0)^3)+(2700- **1875**)*(MAX(gonadotropin dose-3375,0)^3))/(3375-2700))/4100625

gonadotropin dose 3=（MAX(gonadotropin dose- **2250**,0)^3-((3375- **2250**)*(MAX(gonadotropin dose-2700,0)^3)+(2700- **2250**)*(MAX(gonadotropin dose-3375,0)^3))/(3375-2700))/4100625

duration of stimulation 1=（MAX(duration of stimulation-**7**,0)^3-((16-**7**)*(MAX(duration of stimulation-13,0)^3)+(13-**7**)*(MAX(duration of stimulation-16,0)^3))/(16-13))/81

duration of stimulation 2=（MAX(duration of stimulation-**10**,0)^3-((16-**10**)*(MAX(duration of stimulation-13,0)^3)+(13-**10**)*(MAX(duration of stimulation-16,0)^3))/(16-13))/81

duration of stimulation 3=（MAX(duration of stimulation-**11**,0)^3-((16-**11**)*(MAX(duration of stimulation-13,0)^3)+(13-**11**)*(MAX(duration of stimulation-16,0)^3))/(16-13))/81

HMG dose 1=（MAX(HMG dose-**0**,0)^3-((3150-**0**)*(MAX(HMG dose-2475,0)^3)+(2475-**0**)*(MAX(HMG dose-3150,0)^3))/(3150-2475))/9922500

HMG dose 2=（MAX(HMG dose-**825**,0)^3-((3150-**825**)*(MAX(HMG dose-2475,0)^3)+(2475-**825**)*(MAX(HMG dose-3150,0)^3))/(3150-2475))/9922500

HMG dose 3=（MAX(HMG dose-**1987.5**,0)^3-((3150-**1987.5**)*(MAX(HMG dose-2475,0)^3)+(2475-**1987.5**)*(MAX(HMG dose-3150,0)^3))/(3150-2475))/9922500

duration of HMG 1=（MAX(duration of HMG-**0**,0)^3-((16-**0**)*(MAX(duration of HMG-12,0)^3)+(12-**0**)*(MAX(duration of HMG-16,0)^3))/(16-12))/256

duration of HMG 2=（MAX(duration of HMG-**7**,0)^3-((16-**7**)*(MAX(duration of HMG-12,0)^3)+(12-**7**)*(MAX(duration of HMG-16,0)^3))/(16-12))/256

duration of HMG 3=（MAX(duration of HMG-**11**,0)^3-((16-**11**)*(MAX(duration of HMG-12,0)^3)+(12-**11**)*(MAX(duration of HMG-16,0)^3))/(16-12))/256

starting day FSH 1=（MAX(starting day FSH-**1.02**,0)^3-((9.181-**1.02**)*(MAX(starting day FSH-3.86,0)^3)+( 3.86-**1.02**)*(MAX(starting day FSH-9.181,0)^3))/( 9.181- 3.86))/66.4

starting day FSH 2=（MAX(starting day FSH-**1.78**,0)^3-((9.181-**1.78**)*(MAX(starting day FSH-3.86,0)^3)+( 3.86-**1.78**)*(MAX(starting day FSH-9.181,0)^3))/( 9.181- 3.86))/66.4

starting day FSH 3=（MAX(starting day FSH-**2.53**,0)^3-((9.181-**2.53**)*(MAX(starting day FSH-3.86,0)^3)+( 3.86-**2.53**)*(MAX(starting day FSH-9.181,0)^3))/( 9.181- 3.86))/66.4

trigger day E2 1=（MAX(trigger day E2-**640.9**,0)^3-((8060-**640.9**)*(MAX(trigger day E2-4409,0)^3)+(4409-**640.9**)*(MAX(trigger day E2-8060,0)^3))/(8060-4409))/55043041

trigger day E2 2=（MAX(trigger day E2-**1715**,0)^3-((8060-**1715**)*(MAX(trigger day E2-4409,0)^3)+(4409-**1715**)*(MAX(trigger day E2-8060,0)^3))/(8060-4409))/55043041

trigger day E2 3=（MAX(trigger day E2-**2952**,0)^3-((8060-**2952**)*(MAX(trigger day E2-4409,0)^3)+(4409-**2952**)*(MAX(trigger day E2-8060,0)^3))/(8060-4409))/55043041

trigger day P 1=（MAX(trigger day P-**0.35**,0)^3-((2.1-**0.35**)*(MAX(trigger day P-1.265,0)^3)+(1.265-**0.35**)*(MAX(trigger day P-2.1,0)^3))/(2.1-1.265))/ 3.0625

trigger day P 2=（MAX(trigger day P-**0.67**,0)^3-((2.1-**0.67**)*(MAX(trigger day P-1.265,0)^3)+(1.265-**0.67**)*(MAX(trigger day P-2.1,0)^3))/(2.1-1.265))/ 3.0625

trigger day P 3=（MAX(trigger day P-**0.93**,0)^3-((2.1-**0.93**)*(MAX(trigger day P-1.265,0)^3)+(1.265-**0.93**)*(MAX(trigger day P-2.1,0)^3))/(2.1-1.265))/ 3.0625

dominate follicle 1=（MAX(dominate follicle-**2**,0)^3-((15-**2**)*(MAX(dominate follicle-10,0)^3)+(10-**2**)*(MAX(dominate follicle-15,0)^3))/(15-10))/169

dominate follicle 2=（MAX(dominate follicle-**5**,0)^3-((15-**5**)*(MAX(dominate follicle-10,0)^3)+(10-**5**)*(MAX(dominate follicle-15,0)^3))/(15-10))/169

dominate follicle 3=（MAX(dominate follicle-**8**,0)^3-((15-**8**)*(MAX(dominate follicle-10,0)^3)+(10-**8**)*(MAX(dominate follicle-15,0)^3))/(15-10))/169

oocyte yield 1=（MAX(oocyte yield-**2**,0)^3-((21-**2**)*(MAX(oocyte yield-12.05,0)^3)+(12.05-**2**)*(MAX(oocyte yield-21,0)^3))/(21-12.05))/361

oocyte yield 2=（MAX(oocyte yield-**5**,0)^3-((21-**5**)*(MAX(oocyte yield-12.05,0)^3)+(12.05-**5**)*(MAX(oocyte yield-21,0)^3))/(21-12.05))/361

oocyte yield 3=（MAX(oocyte yield-**8**,0)^3-((21-**8**)*(MAX(oocyte yield-12.05,0)^3)+(12.05-**8**)*(MAX(oocyte yield-21,0)^3))/(21-12.05))/361

Table S2 Coefficients of Lasso models refitted for poor responders.

|  | Lasso all features | Lasso without semen | Lasso semen only |
| --- | --- | --- | --- |
| (Intercept) | 1.035741855 | 1.487096 | -1.510131 |
| Female age | . | . | N/A |
| Male age | . | . | N/A |
| Duration of infertility | 0.027875579 | 0.03016404 | N/A |
| Female primary infertility | . | . | N/A |
| Male primary infertility | 0.050551341 | 0.07607849 | N/A |
| PCOS | . | . | N/A |
| Endometriosis | . | . | N/A |
| Previous IUI attempt | 0.428463837 | 0.4599007 | N/A |
| Female height | . | . | N/A |
| Female weight | . | . | N/A |
| Female BMI | . | . | N/A |
| Male height | -0.008231995 | -0.01083925 | N/A |
| Male weight | . | . | N/A |
| Male BMI | . | . | N/A |
| semen volume | . | N/A | . |
| semen volume1 | . | N/A | . |
| semen volume2 | . | N/A | . |
| semen volume3 | . | N/A | . |
| sperm concentration | . | N/A | . |
| sperm concentration1 | . | N/A | . |
| sperm concentration2 | . | N/A | . |
| sperm concentration3 | . | N/A | . |
| sperm normal morphology | . | N/A | . |
| sperm normal morphology1 | . | N/A | . |
| sperm normal morphology2 | . | N/A | . |
| sperm normal morphology3 | . | N/A | 3.41494E-17 |
| sperm motility | . | N/A | . |
| sperm motility1 | . | N/A | . |
| sperm motility2 | . | N/A | . |
| sperm motility3 | . | N/A | . |
| TMC | . | N/A | . |
| TMC1 | . | N/A | . |
| TMC2 | . | N/A | . |
| TMC3 | . | N/A | . |
| Basal FSH | . | . | N/A |
| Basal LH | . | . | N/A |
| Basal PRL | . | . | N/A |
| Basal E2 | . | . | N/A |
| Basal PRL | . | . | N/A |
| Basal T | . | . | N/A |
| AFC | . | . | N/A |
| Agonist | . | . | N/A |
| nonAgonist | . | . | N/A |
| gonadotropin dose | . | . | N/A |
| gonadotropin dose1 | . | . | N/A |
| gonadotropin dose2 | . | . | N/A |
| gonadotropin dose3 | . | . | N/A |
| duration of stimulation | . | . | N/A |
| duration of stimulation1 | . | . | N/A |
| duration of stimulation2 | . | . | N/A |
| duration of stimulation3 | . | . | N/A |
| HMG dose | . | . | N/A |
| HMG dose1 | . | . | N/A |
| HMG dose2 | . | . | N/A |
| HMG dose3 | . | . | N/A |
| Duration of HMG | . | . | N/A |
| Duration of HMG1 | . | . | N/A |
| Duration of HMG2 | -0.019432261 | -0.03463909 | N/A |
| Duration of HMG3 | . | . | N/A |
| Starting dose | . | . | N/A |
| Starting day FSH | . | . | N/A |
| Starting day FSH1 | . | . | N/A |
| Starting day FSH2 | . | . | N/A |
| Starting day FSH3 | . | . | N/A |
| Starting day LH | . | . | N/A |
| Starting day E2 | . | . | N/A |
| HCG type | . | 2.48365E-06 | N/A |
| trigger day E2 | . | . | N/A |
| trigger day E2 1 | . | . | N/A |
| trigger day E2 2 | . | . | N/A |
| trigger day E2 3 | . | . | N/A |
| trigger day LH | . | . | N/A |
| trigger day P | . | . | N/A |
| trigger day P1 | . | . | N/A |
| trigger day P2 | . | . | N/A |
| trigger day P3 | . | -0.2329723 | N/A |
| dominate follicle | . | . | N/A |
| dominate follicle1 | . | . | N/A |
| dominate follicle2 | . | . | N/A |
| dominate follicle3 | . | . | N/A |
| small follicle | . | . | N/A |
| oocyte yield | -0.673093608 | -0.6914354 | N/A |
| oocyte yield 1 | . | . | N/A |
| oocyte yield 2 | . | . | N/A |
| oocyte yield 3 | . | . | N/A |

“.” indicates the coefficient is reduced in the Lasso model.

“N/A” indicates the feature is not applicable in the model.

Table S3 Model performance in poor responders

|  | **LASSO** | **Xgboost** | **LASSO without semen parameters** | **Xgboost without semen parameters** | **LASSO with semen parameters only** | **Xgboost with semen parameters only** |
| --- | --- | --- | --- | --- | --- | --- |
| Sample size | 783 |  |  |  |  |  |
| Prevalence | 0.21(0.18,0.24) |  |  |  |  |  |
| **Original model** |  |  |  |  |  |  |
| Sensitivity | 0.91(0.85,0.94) | 0.73(0.66,0.79) | 0.95(0.91,0.98) | 0.75(0.68,0.81) | 0.48(0.41,0.56) | 0.31(0.25,0.39) |
| Specificity | 0.24(0.21,0.28) | 0.54(0.5,0.58) | 0.08(0.06,0.11) | 0.52(0.48,0.56) | 0.58(0.54,0.61) | 0.75(0.71,0.78) |
| Positive predictive value | 0.24(0.21,0.28) | 0.3(0.25,0.34) | 0.22(0.19,0.25) | 0.29(0.25,0.34) | 0.23(0.19,0.28) | 0.25(0.19,0.31) |
| Negative predictive value | 0.91(0.86,0.94) | 0.88(0.85,0.91) | 0.87(0.76,0.93) | 0.89(0.85,0.92) | 0.81(0.77,0.84) | 0.8(0.77,0.83) |
| Positive likelihood ratio | 1.2(1.08,1.33) | 1.58(1.38,1.82) | 1.04(0.92,1.17) | 1.57(1.37,1.79) | 1.13(0.96,1.35) | 1.23(1,1.5) |
| Negative likelihood ratio | 0.38(0.23,0.62) | 0.5(0.38,0.65) | 0.58(0.28,1.2) | 0.48(0.36,0.63) | 0.9(0.77,1.06) | 0.92(0.82,1.03) |
| Diagnostic odds ratio | 3.2(1.81,6.05) | 3.17(2.14,4.76) | 1.79(0.82,4.45) | 3.27(2.19,4.95) | 1.26(0.88,1.8) | 1.33(0.89,1.96) |
| Accuracy: | 0.38(0.35,0.42) | 0.58(0.54,0.61) | 0.27(0.24,0.3) | 0.57(0.54,0.6) | 0.56(0.52,0.59) | 0.66(0.62,0.69) |
| AUC | 0.66(0.61,0.7) | 0.69(0.65,0.74) | 0.61(0.56,0.66) | 0.69(0.65,0.74) | 0.53(0.48,0.58) | 0.54(0.49,0.59) |
| **Updated model** |  |  |  |  |  |  |
| Sensitivity | 0.45(0.38,0.53) | 0.39(0.32,0.47) | 0.13(0.09,0.2) | 0.27(0.21,0.34) | NA |  |
| Specificity | 0.76(0.72,0.79) | 0.86(0.83,0.89) | 0.93(0.91,0.95) | 0.9(0.88,0.92) | NA | 0.01(0,0.04) |
| Positive predictive value | 0.33(0.27,0.4) | 0.43(0.35,0.51) | 0.33(0.23,0.45) | 0.42(0.33,0.52) | NA | 1(0.99,1) |
| Negative predictive value | 0.84(0.81,0.87) | 0.84(0.81,0.87) | 0.8(0.77,0.83) | 0.82(0.79,0.85) | NA | 0.4(0.12,0.77) |
| Positive likelihood ratio | 1.88(1.55,2.27) | 2.84(2.25,3.6) | 1.89(1.37,2.6) | 2.77(2.1,3.65) | NA | 0.79(0.76,0.82) |
| Negative likelihood ratio | 0.72(0.63,0.84) | 0.71(0.62,0.8) | 0.93(0.87,0.99) | 0.81(0.74,0.89) | NA | 2.52(0.81,7.86) |
| Diagnostic odds ratio | 2.59(1.78,3.77) | 4.01(2.67,6.03) | 2.02(1.12,3.58) | 3.41(2.15,5.39) | NA | 0.99(0.98,1.01) |
| Accuracy: | 0.66(0.73,0) | 0.76(0.73,0.79) | 0.76(0.73,0.79) | 0.77(0.74,0.8) | NA | 0.21(0.18,0.24) |
| AUC | 0.66(0.61,0.7) | 0.69(0.65,0.74) | 0.61(0.56,0.66) | 0.69(0.65,0.74) | NA | 0.79(0.76,0.82) |
| **Refitted model** |  |  |  |  |  |  |
| Sensitivity | 0.59(0.51,0.66) | 0.51(0.44,0.59) | 0.59(0.51,0.66) | 0.32(0.26,0.4) | NA | 0.37(0.3,0.44) |
| Specificity | 0.78(0.74,0.81) | 0.77(0.74,0.8) | 0.78(0.74,0.81) | 0.87(0.84,0.89) | NA | 0.68(0.65,0.72) |
| Positive predictive value | 0.41(0.35,0.47) | 0.37(0.31,0.44) | 0.41(0.35,0.47) | 0.39(0.31,0.48) | NA | 0.23(0.19,0.29) |
| Negative predictive value | 0.88(0.85,0.9) | 0.86(0.83,0.88) | 0.88(0.85,0.9) | 0.83(0.8,0.86) | NA | 0.8(0.77,0.83) |
| Positive likelihood ratio | 2.63(2.18,3.16) | 2.25(1.86,2.72) | 2.61(2.17,3.13) | 2.44(1.91,3.11) | NA | 1.16(0.96,1.39) |
| Negative likelihood ratio | 0.53(0.44,0.64) | 0.63(0.54,0.74) | 0.54(0.44,0.64) | 0.78(0.7,0.87) | NA | 0.93(0.82,1.06) |
| Diagnostic odds ratio | 4.91(3.36,7.2) | 3.55(2.44,5.18) | 4.86(3.33,7.13) | 3.12(2.04,4.75) | NA | 1.24(0.85,1.81) |
| Accuracy: | 0.74(0.71,0.77) | 0.72(0.69,0.75) | 0.74(0.7,0.77) | 0.75(0.72,0.78) | NA | 0.62(0.58,0.65) |
| AUC | 0.72(0.67,0.77) | 0.69(0.65,0.74) | 0.72(0.67,0.76) | 0.68(0.64,0.73) | NA | 0.52(0.47,0.57) |

All values were presented as mean (95% CI).

The original model, models were constructed from the development data.

The updated model, models were calibrated according to the slope and intercept of the calibration curve.

The refitted model, models were constructed from the development data with poor responders only.

NA, evaluation of the model was not applicable due to the lack of discriminating power.

All features, models with all the variables were included; without semen parameters, models with variables except semen parameters were included; models including only semen parameters

Talbe S4 Net reclassification improvement for the overall cohort and subgroups

| name | All | Primary infertility | Secondary infertility | Normalspermia | Male subfertility | Normal responders | Poor responders | Update model in poor responders | Refitted model in poor responders |
| --- | --- | --- | --- | --- | --- | --- | --- | --- | --- |
| Lasso |  |  |  |  |  |  |  |  |  |
| Additive NRI | 5.20 | 8.72 | 0.28 | 0.15 | 6.71 | 3.82 | 11.73 | 14.74 | 0.61 |
| Absolute NRI | 9.19 | 11.37 | 6.48 | 13.68 | 4.81 | 8.75 | 11.75 | -6.77 | 0.13 |
| Event NRI | -5.20 | -4.27 | -7.04 | -15.48 | 1.57 | -5.79 | -4.27 | 31.71 | 0.61 |
| Non-Event NRI | 10.40 | 12.99 | 7.32 | 15.63 | 5.14 | 9.61 | 15.99 | -16.96 | 0.00 |
| Xgboost |  |  |  |  |  |  |  |  |  |
| Additive NRI | -0.07 | 0.05 | 0.35 | -6.17 | 2.80 | 0.50 | -0.21 | 8.16 | 3.83 |
| Absolute NRI | 0.53 | 1.20 | -0.29 | 3.06 | -1.93 | 0.47 | 0.89 | -0.64 | -3.70 |
| Event NRI | -0.71 | -1.42 | 0.70 | -10.12 | 5.49 | 0.00 | -1.83 | 12.20 | 11.59 |
| Non-Event NRI | 0.64 | 1.47 | -0.35 | 3.95 | -2.69 | 0.50 | 1.62 | -4.04 | -7.75 |
